# Enhanced Visualization of Retinal Microvasculature via Deep Learning on OCTA Image Quality

**DOI:** 10.1155/2021/1373362

**Published:** 2021-06-16

**Authors:** Yishuang Xu, Yu Su, Dihao Hua, Peter Heiduschka, Wenliang Zhang, Tianyue Cao, Jingcheng Liu, Zhenyu Ji, Nicole Eter

**Affiliations:** ^1^Eye Center, Renmin Hospital of Wuhan University, Wuhan, China; ^2^Department of Ophthalmology, University of Muenster Medical Center, Muenster, Germany; ^3^Department of Endocrinology & Metabolism, Renmin Hospital of Wuhan University, Wuhan, China

## Abstract

**Purpose:**

To investigate the impact of denoising on the qualitative and quantitative parameters of optical coherence tomography angiography (OCTA) images of the optic nerve and macular area.

**Methods:**

OCTA images of the optic nerve and macular area were obtained using a Canon-HS100 OCT device for 48 participants (48 eyes). Multiple image averaging (MIA) and denoising techniques were used to improve the quality of the OCTA images. The peak signal-to-noise ratio (PSNR) as an image quality parameter and vessel density (VD) as a quantitative parameter were obtained from single-scan, MIA, and denoised OCTA images. The parameters were compared, and the correlation was analyzed between different imaging protocols.

**Results:**

In the optic nerve area, there were significant differences in the PSNR and VD in all measured regions between the three groups (*P* < 0.0001). The PSNR of the denoised group was significantly higher than that of the other two groups (*P* < 0.0001). The VD in the denoised group was significantly lower than that in the single-scan group in all measured regions (*P* < 0.0001). In the macular area, there were significant differences in the PSNR and VD in all measured regions among the three groups. The PSNR of the denoised group was significantly higher than that of the other two groups (*P* < 0.0001). The VD in the denoised group was significantly lower than that in the single-scan group in all measured regions. The VD around the optic nerve in the denoised group was correlated with that in the single-scan group (*R* = 0.9403, *P* < 0.0001), but the VD in the MIA group was not correlated with that in the single-scan group (*R* = 0.2505, *P* = 0.2076). The VD around the fovea in the denoised and MIA images was correlated with that in the single-scan group (*R* = 0.7377, *P* < 0.0001; *R* = 0.7005, *P* = 0.0004, respectively).

**Conclusion:**

Denoising could provide an easy and quick way to improve image quality parameters, such as PSNR. It shows great potential in improving the sensitivity of OCTA images as retinal disease markers.

## 1. Introduction

Optical coherence tomography angiography (OCTA) is a noninvasive imaging method that offers depth-selective and three-dimensional mapping of the retinal microvasculature without dye injection [1]. OCTA provides microvasculature images with higher contrast and better resolution than with fluorescein angiography; furthermore, it contributes to the quantitative evaluation of retinal microvasculature [2]. Currently, OCTA images and quantitative parameters, such as vessel density (VD), have become important markers for retinal disease, and the detailed information of blood flow it provides has contributed greatly to the early detection of multiple eye diseases [3–5].

The quality of OCTA images is crucial for the accurate interpretation of morphological changes in the retinal vasculature and also affects the quantitative analysis results; therefore, improvement of image quality has become a hot research topic [6, 7]. Several protocols have been used to evaluate the image quality of OCTA, including objective evaluations, such as single strength, contrast-to-noise ratio (CNR), and peak signal-to-noise ratio (PSNR), and subjective retinal expert evaluation [8–11]. Of these, the PSNR is one of the most outstanding and widely used objective parameter of image quality evaluation between different images [11, 12]. Recently, it has been reported that multiple en face image averaging (MIA) techniques were able to increase the PNSR of OCTA images, which indicates an improvement in image quality. This technique allows the extraction of a false positive flow signal from multiple images, which lowers the background noise and enhances the positive signal to anneal discontinuous vessel segments [12, 13]. However, acquiring multiple images requires a longer time, which makes it less practical in clinical applications owing to the higher demand of patients' tolerance [13].

Deep learning has become the most common artificial intelligence (AI) techniques, and it shows great potential for automated data analysis and improved image quality [11]. Numerous paired single-scan OCTA and MIA images were applied to deep learning training to develop an algorithm for denoising images. Therefore, an MIA image could be generated from a single-scan image without acquiring multiple OCTA images by using this algorithm [14]. The OCT-HS100 (Canon, Tokyo, Japan), commercialized in recent years, has built-in software to average multiple OCTA images and acquire denoised images using deep learning. It provides a convenient method for the application of the denoising technique in clinical settings. Although previous studies have reported that denoised images show lower background noise and higher image quality than that of single-scan images of the macular area [11, 14], the influence of denoising using commercially available devices on the qualitative and quantitative parameters of the images of both macular and optic areas has not been investigated. The VD is considered an important marker for the early detection of multiple eye diseases; for example, the VD in the macular area can help detect retinal damage in patients with diabetes and hypertension at an early stage, and the VD in the optic area can help in the early diagnosis of glaucoma [3–5]. Thus, the VD in both macular and optic areas acquired from denoised images needs to be verified as credible data before its application in the clinical setting.

Therefore, in this study, we aimed to evaluate the effect of denoising on the qualitative and quantitative parameters of OCTA images in both macular and optic areas by using OCT-HS100, to elucidate if denoising could provide a promising improvement in image quality and reliable quantitative data, and further facilitate the clinical practice with OCTA images as a retinal disease marker.

## 2. Methods

### 2.1. Subjects

Forty-eight eyes of 48 healthy subjects were prospectively included in this study. Eyes with dense lens opacities, corneal opacities, refractive surgery, or a history of intraocular inflammation were excluded. The optic nerve area was scanned in 27 eyes of 27 patients, and the macular area was scanned in 21 eyes of 21 patients. Informed consent was obtained from each enrolled study patient, and the procedures adhered to the tenets of the Declaration of Helsinki.

### 2.2. Optical Coherence Tomography Angiography Imaging

The OCT-HS100 (Ophthalmic Software Platform RX V4.5) built-in software offers a specialized acquisition mode to repeatedly capture consecutive OCTA cube scans at the same position in a short time and can compound multiple OCTA images into a single high-quality image. In addition, this built-in software offers a “denoised OCTA image” assisted by deep learning. In this study, a 3 × 3 mm^2^ scan was chosen for the macular area exam, and a 4.5 × 4.5 mm^2^ scan was chosen for an optic nerve area exam. OCTA imaging was performed by an experienced examiner, and each patient underwent several scans until five OCTA scans that met the criteria were obtained. Images of poor quality (signal strength < 6, motion artifact score (MAS) of 3 or 4, or segmentation errors) were excluded from the quantitative analysis. An MIA image was obtained by choosing five images and applying the MIA function. A denoised image was obtained by choosing one image and applying the denoised function. The PSNR was also recorded and is expressed as the maximum signal divided by the standard deviation [15]. Images were exported and opened in ImageJ (histogram function). The maximum pixel value and standard deviation of the image luminance were calculated [12]. With the exception of PSNR, all measured values were automatically determined using the manufacturer's software.

### 2.3. Statistical Methods

Data management was performed using Microsoft Excel 2010 software. IBM SPSS Statistics 22 for Windows (IBM Corporation, Somers, NY, USA) was used for all statistical analyses. Continuous parametric variables are presented as means ± standard deviations (SD). Repeated-measure one-way analysis of variance (ANOVA) was used to compare the differences between the PSNR and the VD. Tukey's correction was used for multiple comparisons. Pearson's correlation coefficient was used to analyze the correlation between groups. Statistical significance was set at *P* < 0.05.

## 3. Results

### 3.1. Demographic Information

Forty-eight eyes of 48 subjects were prospectively included in this study. The optic nerve area was scanned in 27 patients, of which 13 were women (48.1%). The mean age of the subjects was 50.49 ± 13.00 years. The macular area was scanned in 21 patients; of these, 10 were women (47.6%), and the mean age was 56.29 ± 14.12 years (Table 1).

### 3.2. OCTA Parameters of the Optic Nerve Area

In the optic nerve area, there were significant differences in the PSNR and VD in all measured regions between the three groups (*P* < 0.0001); details are shown in Table 2.

**Figure 1:**
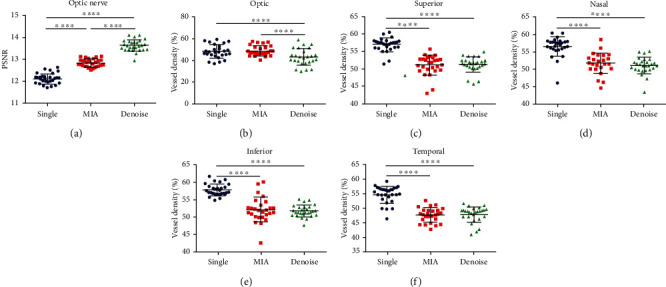
Comparison of three groups in the optic nerve area. Notes: (a) PSNR; (b–f) VD in the optic and superior, nasal, inferior, and temporal sides of the optic nerve. Data are presented as mean ± SD, ^∗∗∗∗^*P* < 0.0001. Abbreviations: PSNR: peak signal-to-noise ratio; MIA: multiple image averaging; VD: vessel density; SD: standard deviation.

Upon further comparison between the two groups, the PSNR of the denoised group was significantly higher than that of the other two groups (*P* < 0.0001). As for the VD, the denoised group had a significantly lower value than the single-scan group in all measured regions (*P* < 0.0001), and only a statistically significant decrease in the optic region was observed when comparing the denoised and MIA groups (*P* < 0.0001) (Table 3 and Figure 1).

**Figure 2:**
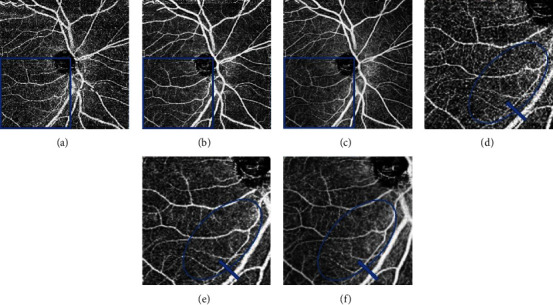
OCTA 4.5 × 4.5 mm^2^ representative images in the optic nerve area of the same subject. Notes: (a, d) single-scan group, (b, e) MIA group, and (c, f) denoised group. (c) shows obviously less background noise and smoother vessels compared to those of (a) and (b), especially on the nasal side. The enlargement of the inferior temporal side of the optic nerve shows a radial peripheral capillary (d) (blue circle), which appeared vague in the MIA group (e) and disappeared in the denoised group (f). Abbreviations: OCTA: optical coherence tomography angiography; MIA: multiple image averaging.

A comparison of the optic area image between the three groups showed that the denoised image exhibited lower background noise and smoother vessels compared to the MIA and single-scan images, especially on the nasal side. A radial peripheral capillary was observed in the enlargement of the inferior temporal side of the optic nerve in a single-scan image, and it became vague in the MIA image and disappeared in the denoised image (Figure 2).

### 3.3. OCTA Parameters of the Macular Area

In the macular area, there were significant differences in the PSNR and VD in all measured regions between the three groups; the details are shown in Table 4.

**Figure 3:**
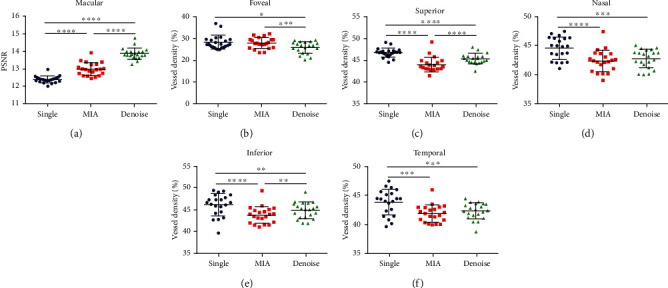
Comparisons of the three groups in the macular area. Notes: (a) PSNR; (b–f) VD in the foveal region and superior, nasal, inferior, and temporal side of the macula. Data are presented as mean ± SD, ^∗^*P* < 0.05, ^∗∗^*P* < 0.01, ^∗∗∗^*P* < 0.001, and ^∗∗∗∗^*P* < 0.0001. Abbreviations: PSNR: peak signal-to-noise ratio; MIA: multiple image averaging; VD: vessel density; SD: standard deviation.

Upon further comparison between the two groups, the PSNR of the denoised group was significantly higher than that of the other two groups (*P* < 0.0001). The VD of the denoised group was significantly decreased compared to that of the single-scan group in all measured regions. Interestingly, the VD in the denoised group was significantly lower in the foveal region compared to that of the MIA group (*P* = 0.0001), but was higher in the superior and inferior regions (*P* < 0.0001, *P* = 0.0089) (Table 5 and Figure 3).

**Figure 4:**
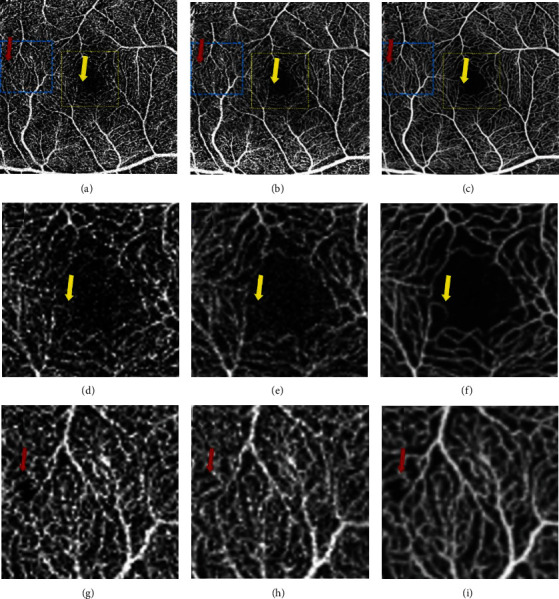
OCTA 3 × 3 mm^2^ representative images on the macular area of the same subject. Notes: (a, d, g) single-scan group, (b, e, h) MIA group, and (c, f, i) denoised group. In the enlargement of the avascular zone (d–f), (f) shows obviously less background noise and smoother vessels compared with those of (d) and (e), and a vessel dropped out in the MIA group and enhanced in the denoised group (yellow arrow); enlargement of the temporal side shows a black shadow covering the blood flow signal in the single-scan group (g) (red arrow), which recovered in the MIA group (h), and persisted in the denoised group (i). Abbreviations: OCTA: optical coherence tomography angiography; MIA: multiple image averaging.

Comparing the macular area image between the three groups showed that the denoised images exhibited lower background noise and smoother vessels compared to the MIA and single-scan images. In the enlargement of the avascular zone, a vessel dropped out in an MIA image and was enhanced in the denoised image, and enlargement of the temporal side showed a black shadow covering the blood flow signal in a single-scan image, which recovered in the MIA image, and persisted in the denoised image (Figure 4).

### 3.4. Correlation Analysis

**Figure 5:**
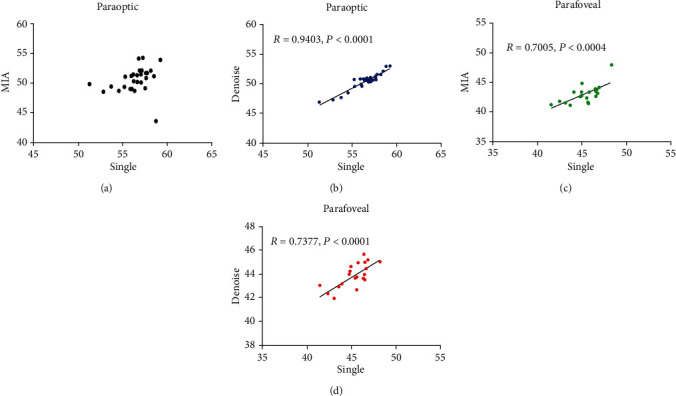
Correlation of the vessel density between two groups. Notes: (a, b) correlation of vessel density in the paraoptic area; (c, d) correlation of vessel density in the parafoveal area.

The VD around the optic nerve in the denoised group was correlated with that in the single-scan group (*R* = 0.9403, *P* < 0.0001), but the VD of the MIA group was not correlated with that of the single-scan group (*R* = 0.2505, *P* = 0.2076). The VD around the foveal region in the denoised and MIA images were both correlated with that in the single-scan group (*R* = 0.7377, *P* < 0.0001; *R* = 0.7005, *P* = 0.0004, respectively) (Figure 5).

## 4. Discussion

OCTA is a noninvasive and fast tool to exhibit retinal microvasculature and has been widely applied in patients with optic and retinal diseases [1]. Morphological changes in retinal vascular and quantitative parameters, including vessel density in the OCTA images, have become an outstanding disease marker in the diagnosis and treatment of optic and retinal diseases. Recent studies have focused on the improvement of OCTA image quality and accordingly increase its sensitivity as a disease marker using different techniques. Among them, AI techniques have shown great prospects [6, 7, 11]. However, research on the application of AI to improve the OCTA image quality was limited in each research group, and the manufacturer's built-in AI denoising function has not been evaluated in the macular and optic nerve areas for practical clinical use.

Image quality has been evaluated objectively and subjectively in past researches. Subjective assessment is easily affected by varies reasons, such as the level of retinal expertise [9]. Objective assessment includes several protocols, such as single strength, CNR, and PSNR, of which the PSNR is one of the most outstanding and widely used objective parameter of image quality evaluation in different images [8, 10–12]. The PSNR is defined as the maximum signal divided by the standard deviation. The PSNR is generally used for a project between the maximum signal and the background noise. Higher PNSR value represents smaller distortion, indicating a higher image quality. Usually after image compression, the output image will be different from the original image to some extent. While evaluating the image quality after processing, the PSNR is usually used to ascertain whether a certain processing procedure is satisfactory. Therefore, in our study, the PNSR was an ideal parameter to compare image quality between the original image and differently processed image [15]. In this study, we compared the impact of two different methods: denoising and MIA on OCTA image quality and VD in both macular and optic nerve areas. Our results showed that the PSNR was significantly higher in the denoised group than in the MIA and single-scan groups, indicating that denoising can improve OCTA image quality even better than MIA by reducing the background noise within a shorter time in both the optic nerve and the macular area. Previous studies also demonstrated better image quality in denoised images than in MIA images [11], which may be because the images used for image averaging have relatively low image quality. Thus, a well-trained denoised algorithm, which outputs high-contrast images, improves the image quality to a greater extent than image averaging. We observed the enhancement of low signal flow in the denoised group, which was eliminated by image averaging (Figures 4(d)–4(f)). This also indicates a more accurate vessel exhibition in the denoised group than in the MIA group, which could improve the diagnosis of early retinal vasculature diseases, such as diabetic retinopathy.

In our study, a significantly lower VD was observed after denoising compared to single-scan OCTA images in both the macular and optic nerve areas. A previous study reported that the VD decreased significantly after MIA, which is in accordance with our results [11]. The denoising algorithm was developed by applying paired single-scan and MIA OCTA images in deep learning training [14]. Eventually, a denoising algorithm was built to generate a similar averaged OCTA image from a single image. This may explain the decrease in VD in both MIA and denoised OCTA images. Using the denoising technique and MIA to process OCTA images, different mechanisms can affect VD. On the one hand, a reduction of noise (and an increase of PSNR) can decrease VD. On the other hand, an increase in continuous vessels can increase VD, as well as an enlargement of vessel caliber [16]. The previous study also reported the lower background noise and better continuity of vessels in the denoised image compare to the MIA image [11]. Therefore, the proportion of vessel and none vessel would determine which factor became the stronger one. In our study, VD in the foveal of the MIA image was significantly higher than that of the denoised image. This may due to the low proportion of vessels in the foveal and result in the reduction of noise which shows a stronger effect on VD in the denoised image. Vice versa, VD in the superior and inferior foveal were a relatively high value compared with those in the nasal and temporal sides in our study, and this could result in the enhancement of vessel continuity which became the stronger factor to impact VD in these two areas which explains why the VD in MIA images was lower than that in the denoised images in areas. In addition, the VD in the denoised images was more correlated with that in the single-scan images than that in the MIA images. The MIA records the exact flow signal from several images, whereas the flow signal of the denoised images was only extracted from the original single OCTA scan. This could be the reason that the VD in the denoised images had a higher correlation to a single OCTA scan than that in the MIA images. In general, VD in the denoised image was significantly changed but still related to VD in the single-scan image. With the reduction of noise and increase in the continuous vessel process, VD in the denoised image should represent the real status of the microvasculature in the retina than that in the single-scan image.

Although the denoising function provides a quicker way to greater image quality acquisition, MIA has some advantages that we observed in this study. Artifacts caused by vitreous turbidity and eye rolling were more likely to be eliminated by image averaging than denoising. Heisler et al. also reported the advantages of MIA in reducing motion artifacts [17]. Such artifacts that change with the movement of the eye were unlikely to be exhibited in the exact position in different scans. However, in the denoised images, these artifacts presented as flakes of low signal, which were difficult to distinguish from the nonperfusion area by morphologic features (Figures 4(g)– 4(i), red arrows). This difference between the denoised and MIA images may be because the denoising algorithm only extracts information from a single scan, which cannot recover the flow information covered by vitreous turbidity. In addition, the application of denoising and image averaging seems less effective in the optic nerve area than in the macular area (Figure 2). The radial peripheral capillary area was difficult to exhibit clearly in the three modes mentioned above, which could be because of the small caliber vessels with a discontinuous image exhibiting a spot-like high signal similar to background noise. This could be eliminated by image averaging and denoising, leading to a loss of flow information. This discovery indicates that there are still some limitations in applying the denoising function of the Canon-HS100 OCTA device in optic nerve diseases, since the potential risk of flow signal is missing. Further deep learning training may help address these problems.

The denoising technique provides a convenient way to enhance the OCTA image quality by processing with AI, which could be a useful tool for patients in whom it is difficult to acquire enough qualified OCTA images because of poor visual acuity. For example, patients with age-related macular degeneration and myopia with poor visual acuity and fixation are likely to benefit from this technique. Theoretically, a more precise VD value, which might improve the detection of sensitivity for the changes retinal vasculature disease such as diabetic retinopathy, can be obtained by this denoising process.

Our study has some limitations. First, we only conducted research in a small sample size, which may influence the accuracy of the statistical results. Second, we only used one OCTA device; it would be better to compare multiple devices to acquire more subject data to verify the reliability of our OCTA parameter results.

## 5. Conclusion

In general, denoising provides an easy and quick way to improve image quality, including PSNR, and shows great potential in improving the sensitivity of OCTA images as retinal disease markers. Although this new technique has some limitations at the current stage, additional training should be conducted for further clinical use.

## Figures and Tables

**Table 1:** Demographic information.

	Optic	Macular
Subjects (*N*)	27	21
Eyes (*N*)	27	21
Age (years)	50.49 ± 13.00	56.29 ± 14.12
Gender (F/M)	13/14	10/11

Notes: age is presented as mean ± SD. Abbreviation: F: female; M: male; SD: standard deviation.

**Table 2:** OCTA parameters of the optic nerve area.

Optic nerve	Single	MIA	Denoise	*F*	*P* value^∗^
PSNR	12.09 ± 0.23	12.82 ± 0.18	13.64 ± 0.26	550.00	<0.0001
Optic	48.34 ± 6.20	48.79 ± 4.63	43.39 ± 7.85	33.36	<0.0001
Superior	56.99 ± 1.97	51.14 ± 2.84	51.34 ± 2.20	87.05	<0.0001
Nasal	56.47 ± 2.78	51.65 ± 2.95	51.07 ± 2.41	69.35	<0.0001
Inferior	57.75 ± 1.69	52.07 ± 3.51	51.68 ± 1.69	71.51	<0.0001
Temporal	54.57 ± 2.97	47.62 ± 2.46	47.81 ± 2.65	197.90	<0.0001

Notes: ^∗^repeated-measure ANOVA was used to compare the differences in the PSNR and VD. Abbreviations: OCTA: optical coherence tomography angiography; PSNR: peak signal-to-noise ratio; MIA: multiple image averaging; VD: vessel density; ANOVA: analysis of variance.

**Table 3:** Multiple comparison between groups in the optic nerve area.

Optic nerve		MIA^∗^	Denoise^∗^
PSNR	Single	<0.0001	<0.0001
	MIA		<0.0001
Optic	Single	0.7441	<0.0001
	MIA		<0.0001
Superior	Single	<0.0001	<0.0001
	MIA		0.9338
Nasal	Single	<0.0001	<0.0001
	MIA		0.6178
Inferior	Single	<0.0001	<0.0001
	MIA		0.8377
Temporal	Single	<0.0001	<0.0001
	MIA		0.9011

Notes: ^∗^Tukey correction was applied for multiple comparisons between two groups. Abbreviations: PSNR: peak signal-to-noise ratio; MIA: multiple image averaging.

**Table 4:** OCTA parameters of macular area.

Macular	Single	MIA	Denoise	*F*	*P* value^∗^
PSNR	12.37 ± 0.19	12.98 ± 0.37	13.87 ± 0.33	344.50	<0.0001
Foveal	28.32 ± 3.15	27.80 ± 2.38	25.85 ± 2.67	9.22	0.0035
Superior	46.71 ± 1.05	43.93 ± 1.66	45.3 ± 1.18	63.89	<0.0001
Nasal	44.52 ± 1.92	42.35 ± 1.88	42.74 ± 1.60	25.52	<0.0001
Inferior	46.10 ± 2.52	43.79 ± 1.94	44.88 ± 1.85	21.80	<0.0001
Temporal	43.74 ± 2.20	41.78 ± 1.49	42.24 ± 1.39	18.40	<0.0001

Notes: ^∗^repeated-measure ANOVA was used to compare the differences in the PSNR and VD. Abbreviations: OCTA: optical coherence tomography angiography; ANOVA: analysis of variance; PSNR: peak signal-to-noise ratio; MIA: multiple image averaging; VD: vessel density.

**Table 5:** Multiple comparison between groups in the macular area.

Macular		MIA^∗^	Denoise^∗^
PSNR	Single	<0.0001	<0.0001
	MIA		<0.0001
Foveal	Single	0.6470	0.0151
	MIA		0.0001
Superior	Single	<0.0001	<0.0001
	MIA		<0.0001
Nasal	Single	<0.0001	0.0002
	MIA		0.4388
Inferior	Single	<0.0001	0.0026
	MIA		0.0089
Temporal	Single	0.0001	0.0005
	MIA		0.3090

Notes: ^∗^Tukey correction was applied for multiple comparisons between two groups. Abbreviations: PSNR: peak signal-to-noise ratio; MIA: multiple image averaging.

## Data Availability

Data are available from corresponding authors upon request.
